# Prevalence of suicidal behaviour in adolescents and youth at ultra-high risk for psychosis: A systematic review and meta-analysis

**DOI:** 10.1192/j.eurpsy.2025.2444

**Published:** 2025-04-03

**Authors:** Shi Han Ang, Siddarth Venkateswaran, Mahir Bakulkumar Goda, Kuhanesan N. C. Naidu, Ganesh Kudva Kundadak, Mythily Subramaniam

**Affiliations:** 1 Yong Loo Lin School of Medicine, National University of Singapore, Singapore; 2 Department of Psychological Medicine, National University Hospital, Singapore; 3 Saw Swee Hock School of Public Health, National University of Singapore, Singapore; 4 Department of Psychological Medicine, Yong Loo Lin School of Medicine, National University of Singapore, Singapore; 5 Research Division, Institute of Mental Health, Singapore; 6 Lee Kong Chian School of Medicine, Nanyang Technological University, Singapore

**Keywords:** ultra high risk, first episode psychosis, schizophrenia, suicide, child and adolescent psychiatry

## Abstract

**Background:**

Suicide remains a major risk factor for individuals suffering from schizophrenia and its prodromal state (i.e., Ultra-High Risk for Psychosis). However, less is known about the prevalence of suicidal behaviour among the adolescent and youth UHR population, a demographic vulnerable to the psychosocial and environmental risk factors of suicide. This review aims to synthesise existing literature on the prevalence of suicidal ideation and behaviour in the adolescent and youth at Ultra-High Risk for Psychosis (UHR), and the associations between suicidal behaviour and its correlates.

**Methods:**

The databases PsycINFO, PubMed, Embase, Cochrane Library, Web of Science, and Scopus were accessed up to July 2024. A meta-analysis of prevalence was subsequently performed for lifetime suicidal ideation, lifetime non-suicidal self-injury, lifetime suicidal attempt, and current suicidal ideation. A narrative review was also carried out for the correlates of suicidal behaviour amongst adolescents and youth in the UHR population.

**Results:**

Studies were included in this meta-analysis. Meta-analysis revealed a high prevalence of lifetime suicidal ideation (58%), lifetime non-suicidal self-injury (37%), lifetime suicidal attempt (25%), and current (2 week) suicidal ideation (56%). The narrative review revealed that a personal transition to psychosis and a positive family history of psychosis were associated with suicidal attempts, while depression was associated with both suicidal attempts and suicidal ideation.

**Conclusion:**

The prevalence of suicidal ideation and behaviour among UHR adolescents and youth is high and comparable to that of the general UHR population. Existing measures that mitigate suicide risk in the general UHR population should be adopted for the youth context.

## Introduction

It has been established that suicidal behaviour is highly prevalent in individuals with schizophrenia. Compared to the healthy population, people with schizophrenia are at a 4.5-fold increased risk of dying from suicide [1], with an estimated rates of 5.6% for completed suicide [2], 20.3% for suicidal attempts [3] and 34.5% for suicidal ideation [4]^.^ This risk is further heightened in the early stages of illness, with up to 40% of total suicides associated with schizophrenia occurring during the First Episode of Psychosis (FEP) [5]. This has given rise to increased clinical focus on individuals experiencing the prodromal stage of psychosis.

Clinicians have characterised this demographic as being at Ultra-High Risk for Psychosis (UHR). UHR individuals are identified by one or more of the following characteristics: (1) Attenuated Psychotic Symptoms (APS); sub-threshold positive psychotic symptoms during the past 12 months; (2) Brief Limited Intermittent Psychotic Symptoms (BLIPS) – frank psychotic symptoms for less than 1 week which resolve spontaneously; and (3) Genetic vulnerability (Trait) – meeting the criteria for Schizotypal Personality Disorder or having a first-degree relative with a psychotic disorder [6].

However, there is a lacuna in the current literature surrounding suicidal behaviour among UHR youths. Most papers have focused on suicide in the general UHR population, with a 2014 meta-analysis establishing a lifetime prevalence of 66% for current suicidal ideation, 18% for lifetime suicide attempts, and 49% for lifetime self-harm behaviour [7]. Yet, youths and adolescents make up most of the UHR population, with only 15% of this demographic aged 25 and above [8]. Furthermore, youth is an inherent risk factor for suicide in the schizophrenia population, with younger patients experiencing higher rates of suicidal ideation and suicidal attempts than their older counterparts [9]. This underscores the need for accurate characterisation of suicidal behaviour and ideation among the UHR youth to provide targeted support for this particularly vulnerable demographic.

The primary aim of this study is to synthesise the existing literature on the prevalence of suicidal ideation and behaviour in the adolescent and youth at Ultra-High Risk for Psychosis (UHR) and provide a meta-analysis on the prevalence of suicidal behaviour and self-harm when appropriate. The secondary aims include comparing the prevalence of suicidal behaviour between UHR and Non-UHR Criteria-fulfilling/Healthy Control (HC)/First Episode Psychosis (FEP) population, and systematically reviewing the risk factors and correlates of suicidal behaviour within the UHR adolescent and young adult population.

## Methods

### Search strategy

**Figure 1.**
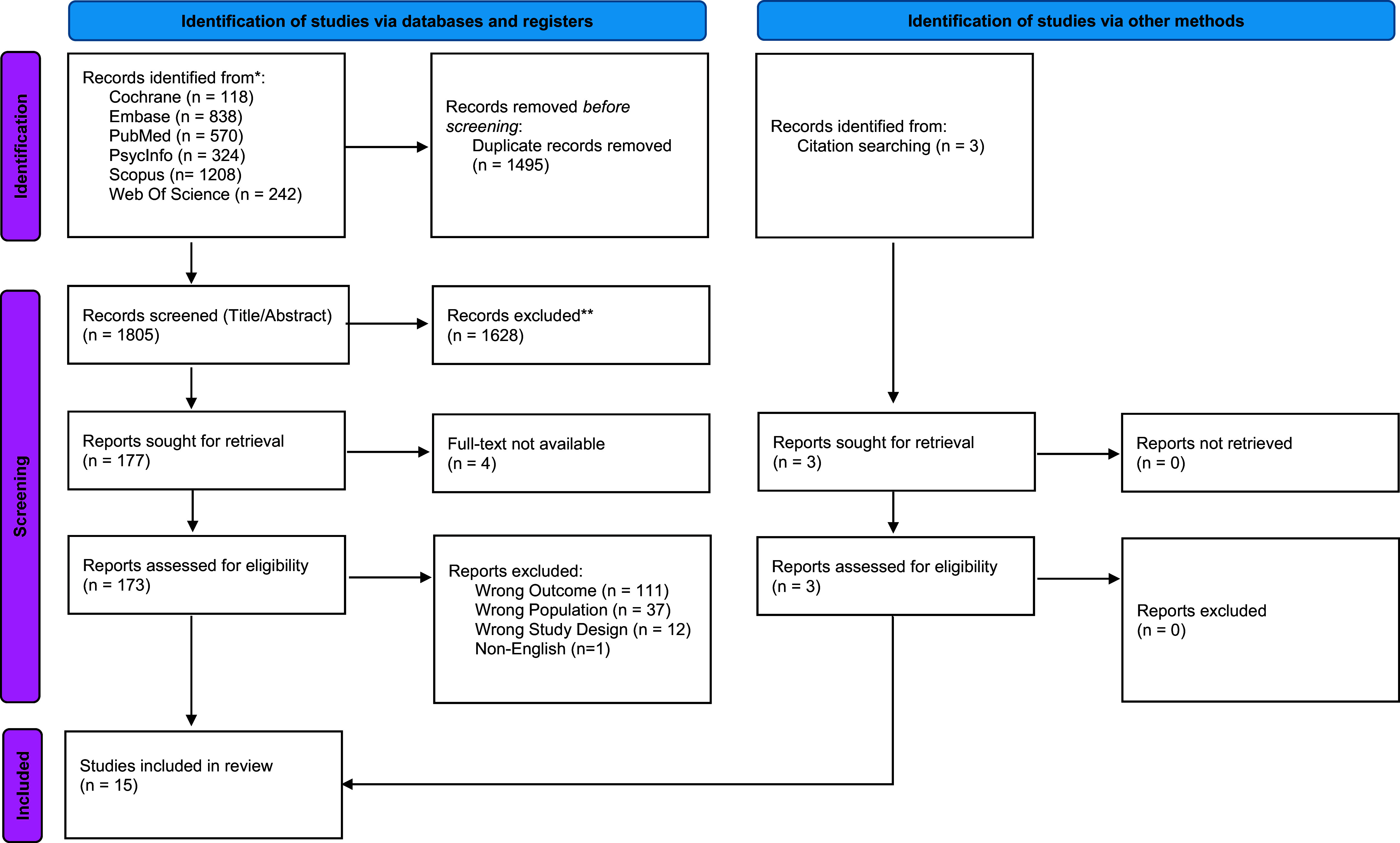
Preferred reporting items for systematic reviews and meta-analyses (PRISMA) flowchart outlining the study selection process.

This meta-analysis was conducted following the MOOSE (Meta-analyses of Observational Studies in Epidemiology) guidelines [10]. (Supplementary Appendix 1) The protocol was registered on PROSPERO: CRD42024583255.) The databases PsycINFO, PubMed, Embase, Cochrane Library, Web of Science, and Scopus were searched from inception up to 31 July 2024. Keywords and controlled vocabulary used consisted of: (“Ultra-High Risk” OR “At Risk Mental State” OR “Clinical High Risk”) AND (“Schizophrenia” OR “Psychosis”) AND (“Self-Harm” OR “Suicide” OR “NSSI”) AND (“Adolescent” OR “Youth”). (Supplementary Appendix 2 – Search strategy. Supplementary Appendix 3 – PICO table.) Title/abstract and full-text screening were conducted by three independent reviewers, with any conflicts resolved by a fourth reviewer. Conference abstracts and theses that were identified through systematic searching were also followed up with the original authors for the full text, if available. Hand-searching was also undertaken within eligible articles to identify suitable articles. Fifteen eligible articles were eventually identified and presented in a PRISMA flow chart (Figure 1).

The inclusion criteria for articles were as follows: studies published in English; participants aged < =25 years; participants classified as UHR according to a validated tool, for example, the Comprehensive Assessment of At-Risk Mental States (CAARMS) [11], the Structured Interview for Psychosis-Risk Symptoms (SIPS) [12], and Prodromal Screen for Psychosis (PROD) [13]; and studies that provided quantitative data on suicidal behaviour and self-harm. Articles that were not written in English, included participants aged over 25, included participants with an established diagnosis of schizophrenia or intellectual disability, history of frank psychotic episodes and extended use of antipsychotics were excluded. The cut-off age of 25 was selected to capture health outcomes of transitional aged youths – a demographic at increased risk of mental illness due to the changes in social roles, peer support, and education that accompany adulthood [14].

In this study, suicidal ideation was defined as the act of thinking about or formulating plans for suicide [15]. Suicidal attempts were defined as self-injurious behaviour done with at least the partial aim of ending one’s life [16]. Non-suicidal self-injury was defined as the intentional destruction of one’s own body tissue without suicidal intent and for purposes that are not socially sanctioned [17]. The term suicidality was defined as the full spectrum of suicidal phenomena, from suicidal ideation to execution [18]. However, it should be acknowledged that the term “suicidality” is controversial among suicidologists due to its lack of precision [19] and will be used in this review only in the context of specific nomenclature (e.g., CAARMS [11], SIPS [12]). It should also be highlighted that non-suicidal self-injury would not fall under the definition of suicidality [20].

### Data extraction

Data extraction commenced on 15 September 2024. Three medical students (A.S.H., S.V., and M.G.) independently undertook data extraction of the predetermined relevant outcomes. Any disagreements between the reviewers were resolved through discussion with a fourth reviewer (G.K.K.), an academic psychiatrist. The authors of one study [21] were contacted for information regarding their demographic breakdown that was missing in the original article, which was later obtained.

### Quality assessment

The methodological quality of the studies included was assessed independently by two authors using the Newcastle-Ottawa Scale (NOS) [22] (Table 1). Studies were considered representative of the exposed cohort if participants were selected from national, state-wide, or regional cohorts. Sufficient follow-up was defined as 6 months or more with an attrition rate of less than 10%. The quality of the articles was classified based on the score obtained into one of the following three and ranked: High (7–9), Medium (5–6), and low (0–5). Among the included studies, 5 were considered high quality, while the remaining 10 studies scored 6 and below. The mean score of the articles was 6.1. However, it should be noted that more than half of the studies were considered cross-sectional and lost a point under the “adequacy of follow-up” criteria due to their study design. Hence, the NOS may underestimate the methodological quality of these studies.

**Table 1.** Newcastle-Ottawa scale

Author, year	Selection				Comparability		Outcome			Total (9/9)
					Comparability of cohorts					
	Representative of exposed cohort	Selection of external control	Ascertainment of exposure	Outcome of interest not present at the start of the study	Main factor	Additional factor	Assessment of outcomes	Sufficient follow-up time	Adequacy of follow-up	
D’Angelo et al. (2017) [31]	*	*	*	*	*	*	*		NA	7/9
Gill et al. (2015) [30]	*		*	*	*	*	*	*		7/9
Grano et al. (2011) [27]		*	*	*	*		*		NA	6/9
Granö et al. (2013) [28]		*	*	*	*		*		NA	6/9
Haining et al. (2020) [35]	*	*	*	*	*	*	*		NA	7/9
Hutton et al. (2011) [33]			*	*	*	*	*			5/9
Kang et al. (2012) [39]		*	*	*	*		*		NA	5/9
Koren et al. (2017) [21]	*		*	*		*	*		NA	5/9
Lindgren et al. (2015) [29]		*	*	*			*	*	*	6/9
Monducci et al. (2024) [38]		*	*	*	*	*	*		NA	6/9
Pelizza et al. [36]	*	*	*	*	*	*	*	*		8/9
Pelizza et al. [37]	*	*	*	*	*	*	*	*	*	9/9
Rasmussen et al. (2019) [40]	*	*	*	*		*	*			6/9
Wastler et al. (2023) [32]	*			*			*	*		4/9
Welsh and Tiffin (2023) [34]	*		*	*			*	*	NA	5/9

* indicates met criteria. NA indicates cross-sectional study design.

A key problem in the methodology not measured by the NOS was the measurement of suicidal behaviour and self-harm. Suicidal behaviour and self-harm were often determined with single self-report items such as the Beck Depression Inventory-II (BDI-II) [23] or continuous subscales measures of suicidality such as the CAARM [11] or SIPS [12]. These scales were developed as one-off measurements and may provide a limited coverage of suicidal behaviour [24]. Nonetheless, it should be noted that the BDI-II has been validated as a strong predictor of the likelihood of patients dying by suicide [25]. Another limitation in the methodology of included studies is the lack of blinding of interviewers to the participants’ UHR status. This may have introduced bias where pre-conceived notions of UHR individuals influenced interviewer perception [26]. Lastly, confounding variables were not consistently applied in studies that analysed correlates of self-harm and suicide. This may lead to biased group comparisons.

### Statistical analysis

A meta-analysis of prevalence was used to estimate the pooled prevalence of lifetime suicide attempts, suicidal ideation, and non-suicidal self-injury when three or more studies were available. A random-effects model with inverse variance weighting was applied to account for between-study heterogeneity, with proportions logit-transformed for variance stabilisation and back-transformed for interpretability. Results are presented with 95% Confidence Intervals (CI) and assessed for heterogeneity using the *I*^2^ statistic. Analyses were performed in RStudio Version 2023.09.1, with statistical significance set at *p* < 0.05. For group comparisons on suicidal behaviour and ideation between UHR and other demographics, the odds ratio was calculated using MedCalc-based population data from the dataset.

## Results

Of the 15 studies selected, seven were longitudinal, while eight were cross-sectional. (Table 2) (Supplementary Appendix 4 – full list of studies included) Three studies were conducted in Finland [27–29], the US [30–32], the UK [33–35], and Italy [36–38] while one study each was conducted in South Korea [39], Israel [21], and Australia [40]. The Comprehensive Assessment of At-Risk Mental State assessment tool (CAARMS) [11] was used most frequently by the studies to evaluate the presence of Ultra-High Risk status in the subjects. Other assessment tools used included the Structured Interview for Prodromal Symptoms (SIPS) [41], Structured Interview for Prodromal Symptoms—Version A (SPI-A) [42], and the Prodromal Questionnaire [43].

**Table 2.** List of included studies

Author, year, country	Study design	Data source	Number and characteristics of participants		Ultra-high risk measuring tool	Outcome measures
			Ultra-high risk	Comparison		
D’Angelo et al. (2017) [31] United States	Cross-sectional	Community	*N* = 40 (20 female); age mean (s.d) = 12.77 (2.77)	*N* = 25 (8 female) psychotic disorder; age mean (s.d) = 12.0 (2.96)	SIPS	SBQ-R: Lifetime suicide attempt and lifetime suicidal ideation
Gill et al. (2015) [30]United States	Longitudinal	Center of Prevention and Evaluation (COPE), New York	*N* = 42 (12 female); age mean (s.d) with suicide ideation = 20.4 (3.4); age mean (s.d) without suicide ideation = 20.2 (4.1)	–	SIPS	C-SSRS: Lifetime SI and Current SI
Granö et al. (2011) Finland [27]	Cross-sectional	Jorvi Early psychosis Recognition and Intervention (JERI) project, Helsinki University Central Hospital, Jorvi Hospital	*N* = 43 (28 female); age mean (s.d) = 14.7 (1.66)	*N* = 37 (16 female) not at risk for psychosis; age mean (s.d) = 14.7 (1.66)	PROD	BDI-II: Current suicidal Ideation
Granö et al. (2013) [28] Finland	Cross-sectional	Jorvi Early psychosis Recognition and Intervention (JERI) project (2009–2011), Helsinki University Central Hospital (HUCH)	*N* = 66 (45 female); age mean (s.d) = 15.6 (2.1)	*N* = 137 (65 female) not at risk for psychosis; age mean (s.d) = 15.2 (2.1)	SIPS	BDI-II: Current suicidal ideation
Haining et al. (2020) [35] United Kingdom	Cross-sectional	Youth Mental Health Risk and Resilience (YouR) study	*N* = 130 (94 female); age mean (s.d) = 21.64 (4.27)	*N* = 15 (10 female) FEP; age mean (s.d) = 21.64 (4.27) *N* = 47 (30 female) psychiatric co-morbids; age mean (s.d) = 22.94 (3.36) *N* = 53 (36 female) HC; age mean (s.d) = 22.42 (3.36)	CAARMS, SPI-A	MINI: Lifetime suicide attempt/suicidality and suicidal ideation
Hutton et al. (2011) [33] United Kingdom	Longitudinal	Salford Early Detection and Intervention Team (EDIT)	*N* = 34 (9 female); age mean (s.d) = 22 (4.6)	–	CAARMS	BDI-II: Current Suicidal Ideation Interview: Lifetime suicide attempt and NSSI
Kang et al. (2012) [39] South Korea	Cross-sectional	Community	*N* = 15 (3 female); age mean (s.d) = 16.8 (0.4)	*N* = 125 (95 female) non-clinical; age mean (s.d) = 16.9 (0.3) *N* = 46 (37 female); age mean (s.d) = 16.7 (0.5)	CAARMS	BDI-II: Current Suicidal Ideation
Koren et al. (2017) Israel [21]	Cross-sectional	Israel Survey of Mental Health among Adolescents (ISMEHA)	*N* = 12 (10 female); age mean (s.d) = 13.9 (0.7)	*N* = 88 (53 female) HC; age mean (s.d) = 14.0 (0.9)	Prodromal Questionnaire, SIPS	K-SADS-PL: Lifetime suicide attempt, Active/Passive Suicidal Attempt, and History of NSSI
Lindgren et al. [29] (2015) Finland	Longitudinal	Helsinki Prodromal Study	*N* = 54 (44 female); age mean (s.d) = 16.7 (0.85)	*N* = 107 (83 female) Non-CHR; age mean (s.d) = 16.6 (0.85)	SIPS	BDI-II: Current suicide ideation, Chart review: Lifetime suicidality
Monducci et al. (2024) [38] Italy	Cross-sectional	Child and Adolescent Neurology and Psychiatry Department of the University-Hospital Policlinico Umberto I and “Sapienza” University of Rome	*N* = 33 (22 female); age mean (s.d.) = 15.2 (1.48)	*N* = 17 (11 female) FEP; age mean (s.d.) = 16.1 (1.40) *N* = 45 (25 female) Other psychiatric disorders; age mean (s.d.) = 15.4 (1.30)	SIPS	Interview: Suicide ideation and suicidal attempt
Pelizza et al. (2019) [36] Italy	Longitudinal	Reggio Emilia At-Risk Mental States (ReARMS) project	*N* = 40 (24 female); age mean (s.d) = 15.34 (1.6)	*N* = 32 (14 female) FEP; age mean (s.d) = 16.3 (1.59) *N* = 40 (18 female) Non-UHR criteria-fulfilling age mean (s.d) = 15.4 (1.75)	CAARMS	Chart review: Suicide Attempt BDI-II: Suicidal Ideation CAARMS: Suicidality
Pelizza et al. (2023) [37] Italy	Longitudinal	Parma At-Risk Mental States (PARMS)	*N* = 164 (78 female); age mean (age range) = 20 (16.5–23)	–	CAARMS	Interview: Suicide attempt
Rasmussen et al. (2020) [40] Australia	Longitudinal	Self and Neurocognition Study; SANE	*N* = 38 (25 female); age mean (s.d) = 19.4 (2.8)	*N* = 26 (15 female) FEP; age mean (s.d) = 19.9 (2.8) *N* = 33 (24 female) HC; age mean (s.d) = 21.1 (1.9)	CAARMS	Chart review: Self-harm and suicide attempt
Wastler et al. (2023) [32] United States	Longitudinal	Ohio State University Early Psychosis Intervention Centre	*N* = 25 (13 female); age mean (s.d) = 19.24 (2.63)	–	SIPS	Chart review, Interview: Lifetime suicide attempt, lifetime suicidal ideation BDI-II: Suicidal ideation
Welsh and Tiffin (2023) [34] United Kingdom	Cross-sectional	Follow-up of the At-Risk Mental State for Psychosis—FARMS Clinic	*N* = 30 (16 female); age mean (s.d) = 15.8 (1.4)	–	CAARMS	Chart review: Self-harm and suicide attempt

CAARMS, Comprehensive Assessment of At-Risk Mental State; SIPS, Structure Interview for Psychotic-risk Symptoms; SPI-A, Schizophrenia Proneness Instrument-Adult; BDI-II, Beck’s Depression Index-II, K-SADS, Kiddie Schedule for Affective Disorders and Schizophrenia; MINI, Mini-International Neuropsychiatric Interview.

The results for lifetime suicidal attempts, current (2 week) suicidal ideation, lifetime suicidal ideation, and lifetime non-suicidal self-injury are displayed in figure plots. Sensitivity analyses were used to further explore the role of individual studies in contributing to heterogeneity.

### Suicidal attempt

**Figure 2.**
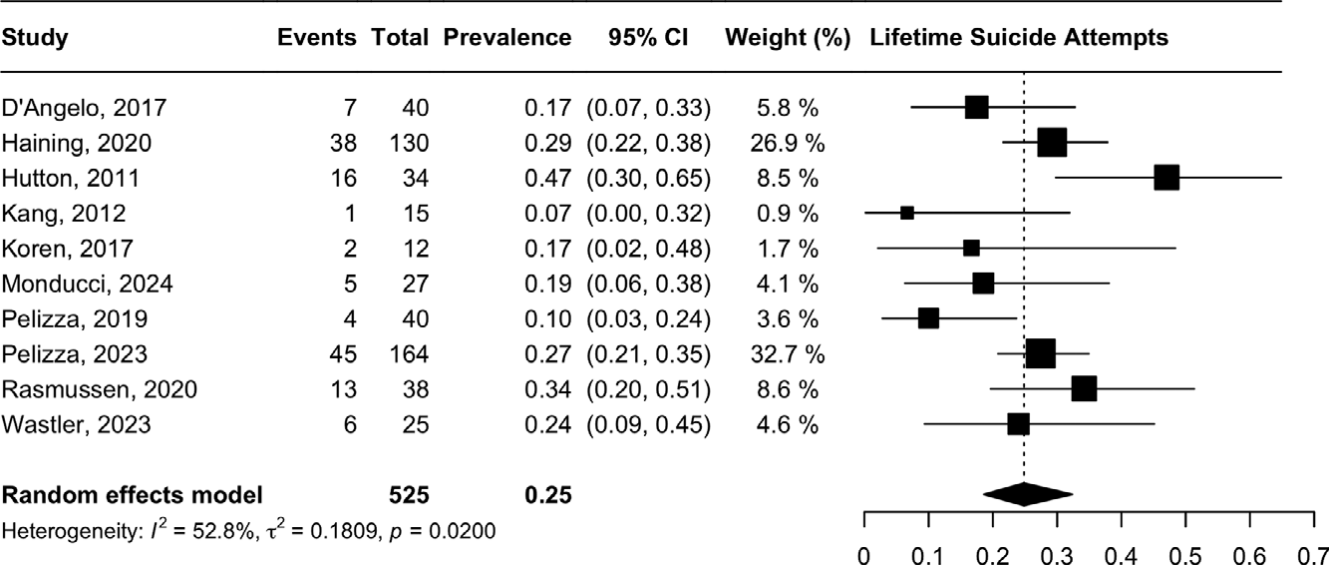
Lifetime suicidal attempt.

The prevalence of lifetime suicide attempts was 24.84% (95% CI 18.6–32.4, *N* = 525, *I*^2^ = 52.8%, p = 0.02), with moderate heterogeneity. (Figure 2.) For past suicidal attempts, one study reported a prevalence of 2.3% (*n* = 3/130) within the past 1 month [35]. Two studies reported longitudinal data on new suicide attempts from the follow-up period. Pelizza et al. [36] reported that 6.25% (*n* = 2/32) and 10.5% (*n* = 2/19) of their cohort had attempted suicide at the 1-year and 2-year follow-up point [36]. Pelizza et al. [37] reported that 7.3% (*n* = 12/164) and 7.9% (*n* = 13/164) of their sample attempted suicide at the 1-year and 2-year follow-up period [37]. However, this figure may be over-represented as some members of the original cohort were unable to be reassessed at the 1- or 2-year mark, as they had withdrawn from the study or were lost to follow-up.

### Current suicidal ideation (2 weeks)

**Figure 3.**
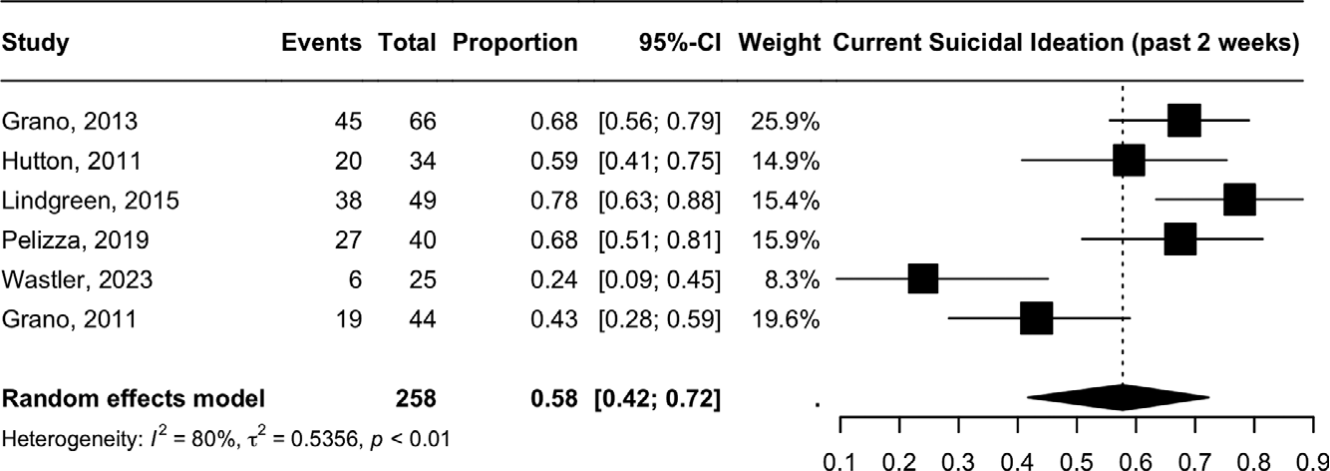
Current suicidal ideation (2 weeks).

Recent (2 week) suicidal ideation had a prevalence of 57.75% (95% CI 41.70–72.31, *n* = 58, *I*^2^ = 80%, *p* = <0.01), with significant heterogeneity. (Figure 3) All studies in the meta-analysis dichotomized the presence and absence of suicidal ideation using the Beck Depression Inventory (BDI-II). The degree of heterogeneity is attributable to the low prevalence reported in Granö et al. [27] (43.18%, *n* = 44) and Wastler et al. [32] (24.00%, *n* = 25). Removal of the following studies resulted in a larger prevalence estimate of 68.43% (95% CI 61.38–74.73) with minor levels of heterogeneity (I = 9.2%, *p* = 0.35).

For the prevalence of SI in the past 1 month, Haining et al. [35] reported the prevalence at 34.6% (*n* = 45/130) [35]. Gill et al. [30] reported the prevalence of suicidal ideation for the past 6 months at 42.9% (*n* = 18/42) [30].

### Suicidal ideation (lifetime)

**Figure 4.**
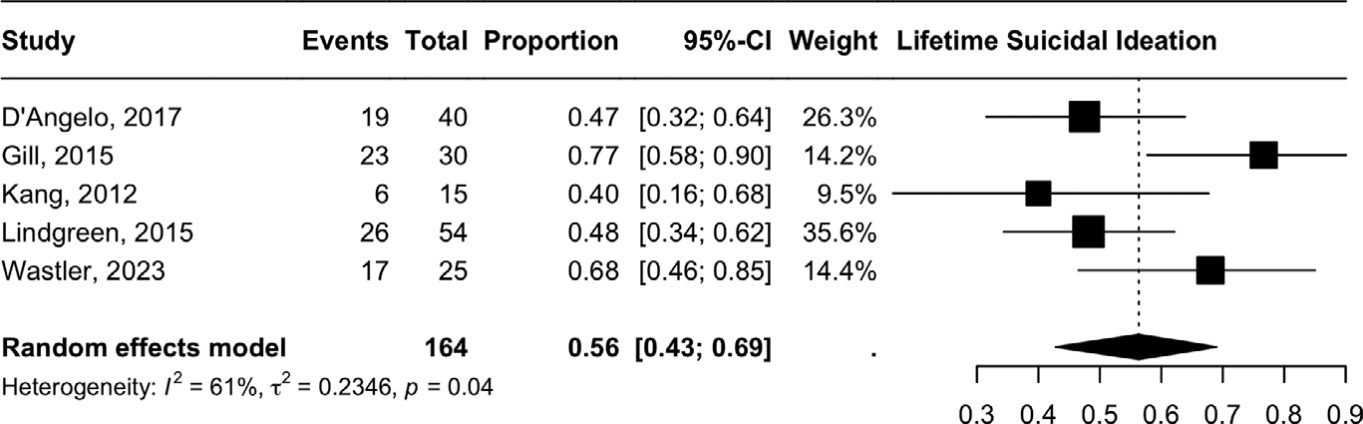
Lifetime suicidal ideation.

The meta-analysis of lifetime suicidal ideation indicated a prevalence of 56.34% (95% CI 42.0–72.0, *n* = 164, *I*^2^ = 61%, *p* = 0.04) with moderate heterogeneity. (Figure 4) The degree of heterogeneity is attributable to the high rates of NSSI reported in Gill et al. [30] (76.77%, *n* = 30) [30]. Excluding this study gave a slightly lower prevalence of 50.49% (95% CI 41.97–58.99) but with lower heterogeneity (*I*^2^ = 22%, *p* = 0.28).

### Non-suicidal self-injury

**Figure 5.**
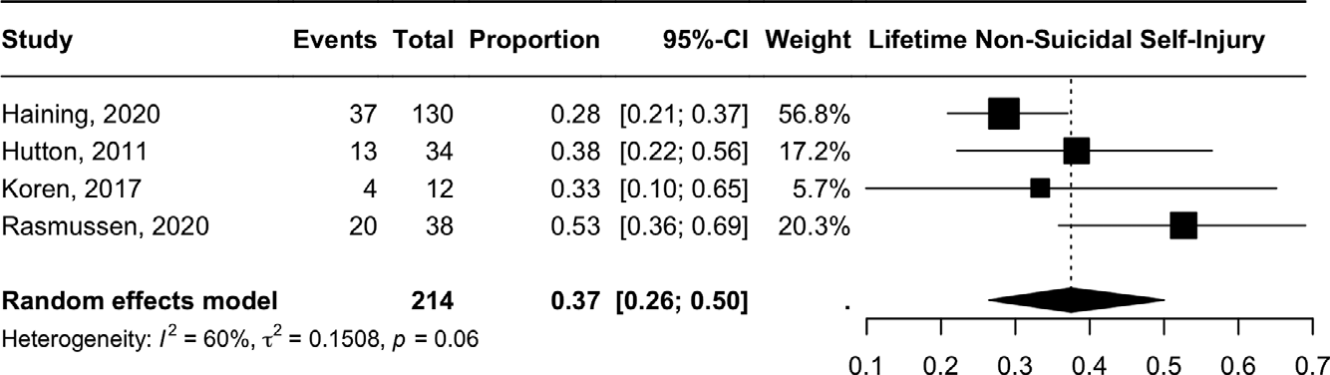
Lifetime non-suicidal self injury.

The meta-analysis of non-suicidal self-injury indicated a prevalence of 37.49% (CI 95% 26.47–49.98, *n* = 214, *I*^2^ = 60%, *p* = 0.060), with moderate heterogeneity. (Figure 5) The degree of heterogeneity is attributable to the high rates of NSSI reported in Rasmussen et al. [40] (52.6%, *n* = 38), whereas the prevalence reported in the other three studies ranges from 28.5 to 38.2%. The removal of this study reduced heterogeneity to non-significant levels (*I*^2^ = 0) and led to a smaller prevalence estimate of 30.79% (CI 95% 24.39–38.03, *p* = 0.54).

For the prevalence of current NSSI (one-month), one study reported it at 5.38% (*n* = 7/130) [35].

### CAARMS/MINI suicidality severity

One study reported continuous mean data for the CAARMS severity scoring, a seven-point scale that reflects the intensity of suicidal thinking and self-harm behaviour. Pelizza et al. [36] reported an average CAARMS suicidality score of 1.83 (95% CI 0.02–3.64) in its population, with 50% (*n* = 20/40) reporting a score of > = 2 [36]. A score of 2 on the CAARMS corresponds to occasional thoughts of self-harm without active suicidal ideation plans [44]. This apparent inconsistency with the high prevalence of suicidal ideation reflected by the BDI-II questionnaire (68.0%, *n* = 27/40) in the same study could be attributed to the interview mode of administration for CAARMS, which might discourage explicit disclosure of suicidal thoughts to the interviewer [45].

Another study reported data on the Mini Neuropsychiatric Interview (MINI) Suicidality Subscale [35]. The MINI Suicidality Subscale categorizes respondents as low, moderate, or high suicidal risk based on six questions relating to recent suicidal ideation, suicidal planning, suicidal attempts, and lifetime suicidal attempts [46]. 21.5% (*n* = 28/130) were classified as low MINI Suicidality risk, while 16.2% (*n* = 21/130) were each classified as moderate and high MINI Suicidality risk. Considering the study’s significant prevalence of past suicidal attempts (29.2%), non-suicidal self-injury (28.5%), and past 1-month suicidal ideation (34.6%), the MINI Suicidality Subscale accurately reflects the high level of suicidality in the studied population.

### Group comparison

Ten studies established comparisons between UHR and other groups (e.g., Non-UHR-Criteria-fulfilling patients, first-episode psychosis, depressive disorders, psychotic disorders, other psychiatric conditions, and healthy control). The large degree of variance by outcome and comparison groups did not allow for a meta-analysis of the results. The results of these comparisons are provided in Table 3.

**Table 3.** Comparison between UHR and other groups

Study, year	Comparison	Outcome	Descriptive statistics	Odds ratio (confidence interval)
Koren et al. (2017) [21]	UHR vs. HC	Current SI	UHR: 5/12 HC: 16/88	3.21 (0.90–11.4) *p* = 0.07
		Lifetime SA	UHR: 2/12 HC: 1/88	**17.4 (1.45–209.5)** ^**a**^
		Lifetime NSSI	UHR: 4/12 HC: 3/88	**14.2 (2.69–74.7)** ^**a**^
Kang et al. (2012) [39]	UHR vs. HC	Lifetime SI	UHR: 6/15 HC: 15/125	**4.89 (1.52–15.7)**
		Lifetime SA	UHR: 1/15 HC: 0/125	**26.0 (1.01–667.33)** ^**a**^
	UHR vs. Depression Spectrum	Lifetime SI	UHR: 6/15 Depression: 31/46	0.32 (0.09–1.07) *p* = 0.06
		Lifetime SA	UHR: 1/15 Depression: 3/46	1.02 (0.09–10.65) *p* = 0.98
Haining et al. (2020) [35]	UHR vs. FEP	Lifetime SA	UHR: 38/130 FEP: 9/15	**0.28 (0.09–0.93)**
		Current SI (past 1 month)	UHR: 45/130 FEP: 11/15	**0.19 (0.06–0.64)**
		Lifetime NSSI	UHR: 37/130 FEP: 9/15	**0.27 (0.09–0.80)**
	UHR vs. Psychiatric Comorbid^b^	Lifetime SA	UHR: 38/130 Psych: 4/47	**4.44 (1.49–13.3)**
		Current SI (past 1 month)	UHR: 45/130 Psych: 9/47	2.24 (0.99–5.03) *p* = 0.0520
		Lifetime NSSI	UHR: 37/130 Psych: 5/47	**3.34 (1.23–9.11)**
	UHR vs. HC	Lifetime SA	UHR: 38/130 HC: 0/53	**44.5 (2.68–740)** ^**a**^
		Current SI (past 1 month)	UHR: 45/130 HC: 1/53	**27.5 (3.68–206)** ^**a**^
		Lifetime NSSI	UHR: 37/130 HC: 2/53	**10.1 (2.35–43.8)** ^**a**^
D’Angelo et al. (2017) [31]	UHR vs. Psychotic disorder	Lifetime SA	UHR: 7/40 Psychotic Disorder: 5/25	0.85 (0.23–3.04) *p* = 0.80
		Lifetime SI	UHR: 19/40 Psychotic Disorder: 18/25	0.35 (0.12–1.03) *p* = 0.0560
Lindgreen et al. (2015) [29]	UHR vs. Non-UHR criteria-fulfilling	Lifetime SI	UHR: 26/54 Non-UHR: 43/107	1.38 (0.72–2.67) *p* = 0.34
		Current SI (past 2 weeks)	UHR: 38/49 Non-UHR: 67/102	1.80 (0.82–3.96) *p* = 0.14
Granö et al. (2013) [28]	UHR vs. Non-UHR criteria-fulfilling	Current SI (past 2 weeks)	UHR: 45/66 Non-UHR: 44/137	**4.53 (2.41–8.50)**
Granö et al. (2011) [27]	UHR vs. Non-UHR criteria-fulfilling	Current SI (past 2 weeks)	UHR: 19/44 Non-UHR: 6/37	**3.93 (1.36–11.3)**
Monducci et al. (2024) [38]	UHR vs. FEP	Current SI (past 2 weeks)	UHR: 16/27 FEP: 5/12	2.04 (0.51–8.10)
Pelizza et al. (2019) [36]	UHR vs. FEP	Lifetime SA	UHR: 7/40 FEP: 2/32	3.18 (0.61–16.5) *p* = 0.17
		Current SI (past 2 weeks)	UHR: 27/40 FEP: 15/32	2.35 (0.90–6.14) *p* = 0.08
		New SA (1-year follow-up)	UHR: 2/32 FEP: 0/24	4.02 (0.18–87.6) *p* = 0.37
		New SA (2-year follow-up)	UHR: 2/19 FEP: 0/11	3.29 (0.14–74.9) *p* = 0.46
	UHR vs. Non-UHR criteria-fulfilling	Lifetime SA	UHR: 7/40 Non-UHR: 1/40	8.27 (0.96–70.7) *p* = 0.0536
		Current SI (past 2 weeks)	UHR: 27/40 Non-UHR: 18/40	**2.54 (1.02 to 6.30)**
		New SA (1-year follow-up)	UHR: 2/32 Non-UHR: 0/31	5.16 (0.24–112.0) *p* = 0.30
		New SA (2-year follow-up)	UHR: 2/19 Non-UHR: 0/10	3.00 (0.13–68.7) *p* = 0.49
Rasmussen et al. (2020) [40]	UHR vs. FEP	Lifetime SA	UHR: 13/38 FEP: 13/26	0.52 (0.19–1.44) *p* = 0.21
		Lifetime NSSI	UHR: 29/38 FEP: 20/26	0.97 (0.30–3.14) *p* = 0.95

Significance = *p* < 0.05, odds ratio (OR) and associated 95% confidence interval calculated from study data for purposes of review. Bolded indicates significant finding.

SI , suicidal ideation; SA , suicide attempt; NSSI , non-suicidal self-injury; HC , healthy control; FEP , first episode psychosis.

a Few cases were present, interpret test and odds ratio with caution.

b Psychiatric comorbid includes mood disorder, anxiety disorder, drug abuse/dependence, alcohol abuse/depending, and eating disorder.

Lifetime suicidal attempts, suicidal ideation, and non-suicidal self-injury were more prevalent among the UHR population compared to healthy controls. Apart from one study [29], current (2 week) suicidal ideation was also higher in UHR groups compared to Non-UHR-Criteria fulfilling groups. Suicidal attempts, suicidal ideation, and non-suicidal self-injury were generally lower in the UHR population compared to the FEP group. There was no significant difference in suicidal behaviour between UHR and groups with Depressive Disorders or Psychotic Disorders.

## Predictors of suicidal behaviour

### Demographics

Two studies reported longitudinal data associating demographic variables and suicide. Pelizza et al. [37] reported a higher prevalence of new suicide attempts in an ethnic (non-Caucasian) population during a 2-year follow-up period, with no associations between gender, age, and education [37]. Girls with UHR status were more likely to be at risk of current suicidal ideation than boys (*p* = 0.008), but this relationship did not hold for lifetime suicidal ideation [29].

### Family history of psychosis

Two studies reported a longitudinal relationship between a family history of psychosis and future suicidal attempts. Having at least one first-degree relative with psychosis was a risk factor for a new suicidal attempt within a 2-year follow-up period (HR = 9.834, *p* < 0.01) [37]. Lingrend et al. [29] reported that a family history of psychosis was also a risk factor for future NSSI in a nine-year follow-up period [29].

### Previous suicide attempts

Haining et al. (2020) reported a positive cross-sectional relationship between previous suicide attempts and lifetime suicidal ideation (OR = 2.701, *p* = 0.040) [35]. Pelizza et al. [37] reported that new longitudinal suicide attempts were associated with a past suicidal attempt (HR = 7.918, *p* = 0.026) [37].

### Transition to psychosis

Two studies reported a longitudinal relationship between eventual transition to psychosis and suicidal behaviour. One study reported that eventual psychosis transition in a 2-year follow-up period strongly predicted a new suicidal attempt (HR = 3.919, *p* = 0.017) [37]. Similarly, psychosis transition within a 9-year follow-up period was associated with new NSSI (Fisher’s exact test *p* = 0.08) [29].

### Psychiatric comorbidity

Psychiatric comorbidity was typically associated with greater suicidal behaviour. Both current and lifetime suicidal ideation were associated with depression (*p* < 0.001, [36]) and non-psychotic mood disorders at baseline (*p* = 0.002 and *p* < 0.001 respectively; [29]). Dysphoric mood (as assessed by SIPS) was also significantly associated with the severity of suicidal ideation. (*r* = 0.52, *p* = 0.001; [31]). Substance usage was found to be related to lifetime suicidal behaviour (Mann–Whitney *U* = 3,387.5, *p* = 0.007; [29]). Co-morbid Axis 1 disorders were also found to be associated with current suicidal ideation in one study (OR = 1.631, *p* = 0.014; [35]); however, details of the specific illnesses investigated were not reported. Anxiety disorder and eating disorder at baseline did not offer predictive value for suicidal behaviour [29].

Certain features of psychosis also exhibited strong associations with suicidal behaviour. Negative symptoms exhibited strong associations with current suicidal ideation (*r* = 0.49, *p* = 0.002; Gill et al., 2015) [30], with one study [29] specifically identifying avolition (*r* = 0.42, *p* < 0.001; [29]) and decreased expression of emotion (*r* = 0.31, *p* < 0.001; [29]) as predictive factors (as measured by SIPS). Basic Self-Disturbance exhibited a strong association with past suicidal attempts [21]. Studies employing continuous subscale measures for UHR psychosis also reported correlations between Huber Basic Symptoms (as measured by CAARMS) and the severity of current suicidal ideation [36]. The “Odd Behaviour/Appearance” subscale of SIPS was also found to be predictive of the severity of lifetime suicidal ideation. (*r* = 0.45, *p* = 0.005; [31]). No association was found between Positive Symptoms and current suicidal ideation [36].

### Functioning

Functional impairment refers to the overall social and occupational impairment caused by psychiatric illness [47]. Functional impairment exhibited strong cross-sectional and longitudinal associations with suicidal behaviour and ideation. Current suicidal ideation was predicted by functional impairment, as measured by decreased Global Assessment Functioning (GAF) (*r* = 0.48, *p* = 0.002; [30]) (*r* = 0.53, *p* = 0.001; [31]) and Global Functioning: Social (GF: Social) scores [35]. New suicidal attempts during a 2-year follow-up period were also predicted by longitudinal functional impairment as measured by CAARMS (HR = 1.70, *p* = 0.02; [37]) School bullying was not found to be a significant predictive factor for suicidal behaviour [29].

### CAARMS severity

Lower CAARMS severity was found to be marginally associated with reduced current suicidal ideation (OR = 0.971, *p* = 0.043; [35]). There was no similar data available for the other validated tools used for UHR Psychosis such as SIPS [12], PROD [38], or K-SADS [48].

## Discussion

The results of this novel meta-analysis suggested that suicidal behaviour was highly prevalent in the UHR youth and adolescent population, particularly with regards to lifetime and current suicidal ideation. Over half of UHR youth reported lifetime (56.34%) and current (57.75%) suicidal ideation, with a quarter (25.00%) reporting a lifetime suicide attempt. A previous meta-analysis on suicidal behaviour in the adult UHR population suggested similar rates of suicidal behaviour (66% prevalence for current suicidal ideation, 18% for lifetime suicide attempts) [7].

Group comparisons between UHR, healthy controls, and First Episode of Psychosis (FEP) groups in this meta-analysis revealed greater lifetime suicidal attempts and suicidal ideation in UHR youth than healthy controls. However, suicidal attempts, suicidal ideation, and non-suicidal self-injury were generally higher in the FEP population than the UHR population. The greater prevalence may be attributed to the difference in psychotic experiences experienced by both demographics. Current literature reflects that both UHR and FEP youth may experience similar levels of impaired social functioning [49] and cognitive dysfunction (e.g., worsening academic performance) [50]. However, the UHR population may be shielded from some of the challenges associated with the first episode of psychosis, including heightened psychotic symptoms [51], distressing interventions such as involuntary hospitalisation [52] and associated stigma [53]. Nonetheless, suicidal behaviour remains a major adverse outcome for UHR youth and should be adequately addressed during intervention.

The risk factors for suicidal behaviour identified in this study mirrors prior findings in the schizophrenia-spectrum disorder population. Co-morbid depression and poor functioning were found to be risk factors in the FEP youth population [54]. Negative symptoms (e.g., anhedonia) were found to be suicidal risk factors in both UHR and the schizophrenia population [55, 56]. Prior suicidal attempts, as a risk factor for new suicidal attempts, was also supported by findings in the FEP youth [57, 58] and general schizophrenia [59] population. This highlights the importance of identifying and treating co-morbidities that drive up the risk of suicide in all stages of psychotic disorders – including UHR, first episode of psychosis, or schizophrenia.

There are certain limitations in this review. Precise definitions for non-suicidal self-injury were not consistently provided by the included studies. This could have led to variances in behaviours that were considered as self-harm between the different studies. These studies could have benefited from utilising standardised nomenclature for defining self-harm [60]. Secondly, studies included in the meta-analysis for current suicidal ideation were limited due to variances in instrumental measurement. The meta-analysis only includes studies that used the BDI-II to assess for current suicidal ideation. This resulted in the exclusion of certain studies that utilised other instruments (e.g., BDI-I [61], C-SSRS [62]). Additionally, studies were too few to allow for systematic exploration of heterogeneity (e.g., publication bias, meta-regression). Nonetheless, heterogeneity was addressed via the random effects model during analysis. The total number of participants for the analyses was also sufficiently large, such that prevalence rates remained high even with the removal of outlier studies. Lastly, language barriers of reviewers also prevented the inclusion of non-English language articles. This may have hindered the generalisability of results in an international context.

In summary, this study demonstrates a concerning level of suicidal behaviour within the UHR youth population, which necessitates a paradigm shift in the treatment of UHR youth. To date, early intervention programmes for UHR youth feature a mix of psychological therapy, pharmacotherapy, family intervention, and social intervention [63]. with the overarching goal of reducing the risk of transition to psychosis [64]. Future emphasis should also be placed on reducing suicidal ideation in this group. Potential psychological treatment methods include Dialectical Behavioural Therapy, which has demonstrated efficacy in reducing adolescent self-harm and suicidal ideation [65]. Increasing the frequency of outpatient follow-up for UHR youth may also reduce suicidal ideation [66]. Recognising the psychological pain – defined as intense feelings of shame, distress and hopeless – associated with UHR psychotic experiences is also important, given its strong predictor of suicidal behaviour [67].

In addition to addressing suicidal behaviour, mental health professionals should also address co-morbidities that increase suicidal risk, such as depression and substance use [68]. Lastly, clinicians working with youths who present with self-harm injuries (e.g., Paediatricians, Emergency Physicians) may also benefit from greater familiarity with the UHR criteria. This allows for early specialist referral and prevents transition to frank psychosis.

## Supporting information

Ang et al. supplementary material Ang et al. supplementary material

## Data Availability

The data that support the findings of this study are available from the corresponding author, A.S.H., upon reasonable request.

## Supplementary material

The supplementary material for this article can be found at http://doi.org/10.1192/j.eurpsy.2025.2444.
