# Supplementary material for: Prevalence of suicidal behaviour in adolescents and youth at ultra-high risk for psychosis: A systematic review and meta-analysis
Source: Eur Psychiatry. 2025 Apr 3;68(1):e56. doi: 10.1192/j.eurpsy.2025.2444 (PMC12090029; doi:10.1192/j.eurpsy.2025.2444)
Supplement: Ang et al. supplementary material [file S0924933825024447sup001.docx]

1. MOOSE Table

**MOOSE (Meta-analyses Of Observational Studies in Epidemiology) Checklist**

A reporting checklist for Authors, Editors, and Reviewers of Meta-analyses of Observational Studies. You must report the page number in your manuscript where you consider each of the items listed in this checklist. If you have not included this information, either revise your manuscript accordingly before submitting or note N/A.

| **Reporting Criteria** | **Reported (Yes/No)** | **Reported on Page No.** |
| --- | --- | --- |
| **Reporting of Background** |  |  |
| Problem definition | Yes | 2 |
| Hypothesis statement | N/A |  |
| Description of Study Outcome(s) | Yes | 3 |
| Type of exposure or intervention used | Yes | 3 |
| Type of study design used | Yes | 3 |
| Study population | Yes | 3 |
| **Reporting of Search Strategy** |  |  |
| Qualifications of searchers (eg, librarians and investigators) | Yes | 5 |
| Search strategy, including time period included in the synthesis and keywords | Yes | 3-4 |
| Effort to include all available studies, including contact with authors | Yes | 4-5 |
| Databases and registries searched | Yes | 3-4 |
| Search software used, name and version, including special features used  (eg, explosion) | Yes | 3-4 |
| Use of hand searching (eg, reference lists of obtained articles) | Yes | 3-4 |
| List of citations located and those excluded, including justification | Yes | See supplement document |
| Method for addressing articles published in languages other than  English | N/A |  |
| Method of handling abstracts and unpublished studies | Yes | 3-4 |
| Description of any contact with authors | Yes | 5 |
| **Reporting of Methods** |  |  |
| Description of relevance or  appropriateness of studies assembled for assessing the hypothesis to be tested | Yes | 5-6 |
| Rationale for the selection and coding of data (eg, sound clinical principles or convenience) | Yes | 5-6 |
| Documentation of how data were classified and coded (eg, multiple raters, blinding, and interrater reliability) | Yes | 5 |
| Assessment of confounding (eg, comparability of cases and controls in studies where appropriate | Yes | 5-6 |
| **Reporting Criteria** | **Reported (Yes/No)** | **Reported on Page No.** |
| Assessment of study quality, including blinding of quality assessors; stratification or regression on possible predictors of study results | Yes | 5-6 |
| Assessment of heterogeneity | Yes | 6 |
| Description of statistical methods (eg, complete description of fixed or random effects models, justification of whether the chosen models account for predictors of study results, dose-response models, or cumulative meta-analysis) in sufficient detail to be replicated | Yes | 6 |
| Provision of appropriate tables and graphics | Yes | See figure document |
| **Reporting of Results** |  |  |
| Table giving descriptive information for each study included | Yes | See figure document |
| Results of sensitivity testing (eg, subgroup analysis) | N/A |  |
| Indication of statistical uncertainty of findings | Yes | 7-9 |
| **Reporting of Discussion** |  |  |
| Quantitative assessment of bias (eg, publication bias) | N/A |  |
| Justification for exclusion (eg, exclusion of non–English-language citations) | Yes | 14-15 |
| Assessment of quality of included studies | Yes | 5-6, 14-15 |
| **Reporting of Conclusions** |  |  |
| Consideration of alternative explanations for observed results | Yes | 14-15 |
| Generalization of the conclusions (ie, appropriate for the data presented and within the domain of the literature review) | Yes | 14-15 |
| Guidelines for future research | Yes | 15 |
| Disclosure of funding source | Yes | 16 |

**Once you have completed this checklist, please save a copy and upload it as part of your submission. DO NOT** **include this checklist as part of the main manuscript document. It must be uploaded as a separate file.**

1. Search Strategy

PubMed

| Keywords (Title/Abstract) | Mesh Terms |
| --- | --- |
| 1 "Clinical High Risk"[Title/Abstract] OR "At Risk Mental State"[Title/Abstract] OR "Ultra High Risk"[Title/Abstract] OR "CAARMS"[Title/Abstract] OR "prodrom*"[Title/Abstract] OR "SIPS"[Title/Abstract] OR "SOPS"[Title/Abstract] OR "Prevent*"[Title/Abstract] | - |
| 2 Schizo*[Title/Abstract] OR Psychosis[Title/Abstract] OR Psychotic[Title/Abstract] | 5 Schizophrenia[MeSH Terms] |
| 3 "Self harm"[Title/Abstract] OR "Suicid*"[Title/Abstract] OR "Self injur*"[Title/Abstract] OR "Self mutilat*"[Title/Abstract] OR "NSSI"[Title/Abstract] OR "Non suicidal self injury"[Title/Abstract] OR “Automutilation”[Title/Abstract] OR “Self mutilation”[Title/Abstract] OR ‘suicid* idea*’[Title/Abstract] | 6 Suicide[MeSH Terms] OR  Self Mutilation[MeSH Terms] |
| 4 "Child*"[Title/Abstract] OR "Teen*"[Title/Abstract] OR "Adolescent Pediatric*"[Title/Abstract] OR "Youth"[Title/Abstract] OR "Young"[Title/Abstract] OR "Young adult"[Title/Abstract] OR "Transitional age youth"[Title/Abstract] | 7 Adolescent[MeSH Terms] OR  Child[MeSH Terms] OR  Young Adult[MeSH Terms] |

1 AND (2 OR 5) AND (3 OR 6) AND (4 OR 7)

Embase

| Keywords (Title/Abstract) | Emtree |
| --- | --- |
| 1 'clinical high risk':ab,ti OR 'at risk mental state':ab,ti OR 'ultra high risk':ab,ti OR 'caarms':ab,ti OR 'prodrom*':ab,ti OR 'sips':ab,ti OR 'sops':ab,ti OR 'prevent*':ab,ti | 5 Ultra high risk for psychosis/exp |
| 2 schizo*:ab,ti OR psychosis:ab,ti OR psychotic:ab,ti | 6 Schizophrenia/exp |
| 3 'self harm':ab,ti OR 'suicid*':ab,ti OR 'self injur*':ab,ti OR 'self mutilat*':ab,ti OR ‘automutilation’:ab,ti OR 'nssi':ab,ti OR 'non suicidal self injury':ab,ti OR ‘suicd* idea*’:ab,ti | 7 Suicide/exp  Automutilation/exp |
| 4 child*:ab,ti OR teen*:ab,ti OR adolescent:ab,ti OR pediatric*:ab,ti OR youth:ab,ti OR young:ab,ti OR adult:ab,ti OR 'transitional age youth':ab,ti | 8 Adolescent/exp  Young adult/exp  Child/exp  Transitional age youth/exp |

(1 OR 5) AND (2 OR 6) AND (3 OR 7) AND (4 OR 8)

Cochrane Library

| Keywords (Title/Abstract) | Mesh Term |
| --- | --- |
| 1 ('clinical high risk' OR 'at risk mental state' OR 'ultra high risk' OR 'caarms' OR 'prodrom*' OR 'sips' OR 'sops' OR 'prevent*'):ti,ab | - |
| 2 (schizo* OR psychosis OR psychotic):ti,ab | 5 Schizophrenia[MeSH Terms] |
| 3 ('self harm' OR 'suicid*' OR 'self injur*' OR 'self mutilat*' OR ‘automutilation’ OR 'nssi' OR 'non suicidal self injury' OR 'suicid* idea*'):ti,ab | 6 Suicide[MeSH Terms] OR  Self Mutilation[MeSH Terms] |
| 4 (child* OR teen* OR adolescent OR pediatric* OR youth OR young OR young adult OR 'transitional age youth'):ti,ab | 7 Adolescent[MeSH Terms] OR  Child[MeSH Terms] OR  Young Adult[MeSH Terms] |

1 AND (2 OR 5) AND (3 OR 6) AND (4 OR 7)

PsycInfo

| Keywords (Title/Abstract) | Psyinfo Thesaurus |
| --- | --- |
| 1 ('clinical high risk' OR 'at risk mental state' OR 'ultra high risk' OR 'caarms' OR 'prodrom*' OR 'sips' OR 'sops' OR 'prevent*).ti,ab | - |
| 2 (schizo* OR psychosis OR psychotic).ti,ab | 5 Schizophrenia/ |
| 3 ('self harm' or 'suicid*' or 'self injur*'OR 'self mutilat*' or 'nssi' or ‘automutilation’ OR 'non suicidal self injury' or 'suicid* idea*').ti,ab. | 6 Nonsuicidal Self-Injury/ OR Suicidal Behavior/ |
| 4 (child* OR teen* OR adolescent OR pediatric* OR youth OR young OR young adult OR 'transitional age youth').ti,ab | 7 Young Adulthood/ OR Early Adolescence/ OR Late Adolescence/ |

1 AND (2 OR 5) AND (3 OR 6) AND (4 OR 7)

Web of Science

| Keywords (Title/Abstract) |
| --- |
| TI=(('clinical high risk' OR 'at risk mental state' OR 'ultra high risk' OR 'caarms' OR 'prodrom*' OR 'sips' OR 'sops' OR 'prevent*')) OR AB=(('clinical high risk' OR 'at risk mental state' OR 'ultra high risk' OR 'caarms' OR 'prodrom*' OR 'sips' OR 'sops' OR 'prevent*')) |
| TI=(schizo* OR psychosis OR psychotic) OR AB=(schizo* OR psychosis OR psychotic) |
| AB=('self harm' or 'suicid*' or 'self injur*'OR 'self mutilat*' or 'nssi' or ‘automutilation’ OR 'non suicidal self injury' or 'suicid* idea*') OR TI=('self harm' or 'suicid*' or 'self injur*'OR 'self mutilat*' or 'nssi' or ‘automutilation’ OR 'non suicidal self injury' or 'suicid* idea*') |
| TI=(child* OR teen* OR adolescent OR pediatric* OR youth OR young OR young adult OR 'transitional age youth') OR AB=(child* OR teen* OR adolescent OR pediatric* OR youth OR young OR young adult OR 'transitional age youth') |

1 AND 2 AND 3 AND 4

Scopus

( TITLE-ABS-KEY ( "clinical high risk" OR "at risk mental state" OR "ultra high risk" OR "caarms" OR "prodrom*"OR "sips" OR "sops" OR "prevent*" ) AND TITLE-ABS-KEY ( schizo* OR psychosis OR psychotic ) AND TITLE-ABS-KEY ( "self harm" OR suicid* OR "self injur*" OR "self mutilat*" OR 'nssi' OR ‘automutilation’ OR "non suicidal self injury" OR "suicid* idea*" ) AND TITLE-ABS-KEY ( child* OR teen* OR adolescent OR pediatric* OR youth OR young OR "young adult" OR "transitional age youth" ) )

1. PICO Table

| PICOS | Inclusion Criteria | Exclusion Criteria |
| --- | --- | --- |
| Population | - Age <=25 yo - Diagnosed with Ultra High Risk (UHR) / At Risk Mental State (ARMS) / Clinical High Risk (CHR) of Psychosis - Validated tool used to assess for UHR e.g. CAARMS (Comprehensive Assessment of At Risk Mental States) | - Population >25 - Diagnosis of Schizophrenia - History of frank psychotic episodes - Extended use of antipsychotic medication - Diagnosis of intellectual disability / ASD |
| Intervention | NA | Nil |
| Comparison | - Healthy Controls - Patients with other Psychiatric illness - e.g. First Episode Psychosis, Depression Spectrum disease etc. | Nil |
| Exposure of interest | - 1. Prevalence of suicide ideation, Non-Suicidal Self-Injury (NSSI), suicide attempt in UHR youth.   2. Potential correlates and predictors of self-harm: e.g. Demographic variables, previous suicide attempts, psychiatric co-morbidity, treatment   3. Suicidality outcomes in comparison with healthy controls/first episode psychosis/other psychiatric diseases etc. | Nil |
| Study design | - Primary studies   - Descriptive papers   - Qualitative, quantitative, and mixed study methods   - Randomised controlled trials, cohort studies, case-control studies, cross-sectional studies - Year: All | - Papers not in English - Secondary systematic reviews - Guidelines, commentaries, viewpoints, editorials, book chapters, letters to editors, conference abstracts, theses and dissertations - Study protocols |
| Geographic Location | Worldwide | - |
| Language | English studies | Non-English studies |
| Sample size | Nil | Nil |
| Paper Completion | Completed papers | Incompleted Papers |

1. List of included studies

D’Angelo EJ, Lincoln SH, Morelli N, Graber K, Tembulkar S, Gonzalez-Heydrich J. Suicidal behaviors and their relationship with psychotic-like symptoms in children and adolescents at clinical high risk for psychosis. Comprehensive Psychiatry. 2017 Oct;78:31–7.

Gill KE, Quintero JM, Poe SL, Moreira AD, Brucato G, Corcoran CM, et al. Assessing suicidal ideation in individuals at clinical high risk for psychosis. Schizophrenia Research. 2015 Jul;165(2-3):152–6.

Granö N, Karjalainen M, Suominen K, Roine M. Poor functioning ability is associated with high risk of developing psychosis in adolescents. Nordic Journal of Psychiatry. 2010 May 14;65(1):16–21.

Granö N, Karjalainen M, Edlund V, Saari E, Itkonen A, Anto J, et al. Depression symptoms in help-seeking adolescents: A comparison between adolescents at-risk for psychosis and other help-seekers. Journal of Mental Health. 2013 Jan 16;22(4):317–24.

Haining K, Karagiorgou O, Gajwani R, Gross J, Gumley AI, Lawrie SM, et al. Prevalence and predictors of suicidality and non‐suicidal self‐harm among individuals at clinical high‐risk for psychosis: Results from a community‐recruited sample. Early Intervention in Psychiatry. 2020 Dec 28;

Hutton P, Bowe S, Parker S, Ford S. Prevalence of suicide risk factors in people at ultra-high risk of developing psychosis: a service audit. Early Intervention in Psychiatry. 2011 Oct 27;5(4):375–80.

Kang NI, Park TW, Yang JC, Oh K, Shim SH, Young Keun Chung. Prevalence and clinical features of Thought–Perception–Sensitivity Symptoms: Results from a community survey of Korean high school students. Psychiatry Research-neuroimaging. 2012 Aug 15;198(3):501–8.

Koren D, Rothschild‐Yakar L, Lacoua L, Brunstein‐Klomek A, Zelezniak A, Parnas J, et al. Attenuated psychosis and basic self‐disturbance as risk factors for depression and suicidal ideation/behaviour in community‐dwelling adolescents. Early Intervention in Psychiatry. 2017 Nov 22;13(3):532–8.

Lindgren M, Manninen M, Kalska H, Mustonen U, Laajasalo T, Moilanen K, et al. Suicidality, self-harm and psychotic-like symptoms in a general adolescent psychiatric sample. Early Intervention in Psychiatry. 2015 Jan 13;11(2):113–22.

Monducci E, Mammarella V, Maffucci A, Michela Colaiori, Cox O, Cesario S, et al. Psychopathological Characteristics and Subjective Dimensions of Suicidality in Adolescents at Ultra High Risk (UHR) for Psychosis. Early Intervention in Psychiatry. 2024 Dec 27;

‌

Pelizza L, Poletti M, Azzali S, Paterlini F, Garlassi S, Scazza I, et al. Suicidal Thinking and Behavior in Adolescents at Ultra‐High Risk of Psychosis: A Two‐year Longitudinal Study. Suicide and Life-Threatening Behavior. 2019 Apr;49(6):1637–52.

Pelizza L, Leuci E, Quattrone E, Azzali S, Pupo S, Paulillo G, et al. Adverse outcome analysis in people at clinical high risk for psychosis: results from a 2-year Italian follow-up study. Social Psychiatry and Psychiatric Epidemiology. 2023 Dec 13;59(7):1177–91.

Rasmussen AR, Reich D, Lavoie S, Li E, Hartmann JA, McHugh M, et al. The relation of basic self‐disturbance to self‐harm, eating disorder symptomatology and other clinical features: Exploration in an early psychosis sample. Early Intervention in Psychiatry. 2019 Jul 2;14(3):275–82.

Wastler HM, Cowan HR, Hamilton SA, Lundin NB, Manges M, Moe AM, et al. Variability in suicidal ideation during treatment for individuals at clinical high risk for psychosis: The importance of repeated assessment. Early Intervention in Psychiatry. 2023 Apr 6;17(10):1038–41.

Welsh P, Tiffin PA. The “At-Risk Mental State” for Psychosis in Adolescents: Clinical Presentation, Transition and Remission. Child Psychiatry & Human Development. 2013 Apr 14;45(1):90–8.
